# The importance and mitigation of mycotoxins and plant toxins in Southeast Asian fermented foods

**DOI:** 10.1038/s41538-022-00152-4

**Published:** 2022-08-31

**Authors:** Iyiola O. Owolabi, Oluwatobi Kolawole, Phantakan Jantarabut, Christopher T. Elliott, Awanwee Petchkongkaew

**Affiliations:** 1 School of Food Science and Technology, Faculty of Science and Technology, Thammasat University, 99 Mhu 18, Phahonyothin Road, Khong Luang, Pathum Thani 12120 Thailand; 2 International Joint Research Center on Food Security (IJC-FOODSEC), 113 Thailand Science Park, Phahonyothin Road, Khong Luang, Pathum Thani 12120 Thailand; 3 Institute for Global Food Security, School of Biological Science, Queen’s University Belfast, 19 Chlorine Gardens Belfast, BT9 5DL Belfast, Northern Ireland

**Keywords:** Food microbiology, Mass spectrometry

## Abstract

Fermented foods (ffs) and beverages are widely consumed in Southeast Asia (SEA) for their nutritional balance, flavor, and food security. They serve as vehicles for beneficial microorganisms performing a significant role in human health. However, there are still major challenges concerning the safety of ffs and beverages due to the presence of natural toxins. In this review, the common toxins found in traditional ffs in SEA are discussed with special reference to mycotoxins and plant toxins. Also, mitigation measures for preventing risks associated with their consumption are outlined. Ochratoxin, citrinin, aflatoxins were reported to be major mycotoxins present in SEA ffs. In addition, soybean-based ff food products were more vulnerable to mycotoxin contaminations. Common plant toxins recorded in ffs include cyanogenic glycosides, oxalates, phytates and saponins. Combined management strategies such as pre-harvest, harvest and post-harvest control and decontamination, through the integration of different control methods such as the use of clean seeds, biological control methods, fermentation, appropriate packaging systems, and controlled processing conditions are needed for the safe consumption of indigenous ffs in SEA.

## Introduction

The process of fermentation is one of the oldest biotechnologies employed in the production of food products with beneficial properties including prolonged shelf-life and satisfactory organoleptic characteristics^1^. Finished products from fermented foods (ffs) generally possess enhanced microbial stability and safety and some can be preserved even at room temperature. In addition, the process of food fermentation proffer food products with improved consumers’ palatability. For these reasons, there has been heightened interest in exploring this natural process and more specifically, to link the heterogeneity of the fermenting microbial community and their properties to the quality of the product^2^. Fermentation is a process used in the production of foods, beverages and other beneficial metabolites either by aerobic or anaerobic microorganisms through the enzymatic conversions of substrates and microbial growth^3^. By sensing the environment, microbes produce signals to trigger protein synthesis (enzymes), while substrates are converted enzymatically into nutrients, such as amino acids, sugars, nucleotides, fatty acids, and vitamins, which are necessary for the growth of the microorganism. Therefore, the activities of microorganisms are significant in food fermentation as they help to prolong the shelf life of foods, enhance the organoleptic, nutritional and antioxidant properties, and aids in the reduction/removal of toxic ingredients from the raw materials^2^.

The history of fermentation can be traced back to the Neolithic period (circa 10,000 B.C.) when it was mainly used for extending the shelf-life of perishable foods. The primary aim of food preservation was obtained through the production of inhibitory growth substances namely, organic acid ethanol, and bacteriocins, combined with a decreased water activity^4^. Therefore, traditional ffs and beverages have been in existence and have been incorporated into the human diet from the onset of civilization. Since then, food fermentation has been widely studied for other purposes including enhancement of food safety by way of suppressing pathogens or elimination of toxigenic compounds; boosting nutritional components, and improving the organoleptic properties of the food^5^. Fermented foods have the potential to tackle the global problem linked to a having a balanced diet as they are embedded with a distinct group of microflora that improves the nutritional components of foods including proteins, essential amino acids, vitamins, and fatty acids^6^. Fermenting organisms are also capable of releasing bioactive metabolites such as peptides, conjugated linoleic acids, exopolysaccharides, neurotransmitters (γ-aminobutyric acids also known as GABA), and oligosaccharides^7^.

In Asia, ffs are important components of human diets and, the international market for fermented ingredients and food products is estimated to rise to $28.4 billion in 2022^8^. In SEA, fermentation of cereal grain to derive different food product varieties have been a tradition for a long time. An example of this is rice wine, a popular alcoholic beverage in Asia. In Malaysia and Indonesia, the fermentation of cassava tubers is carried out to produce the well-known sweet and sour snack known as *tapai/tape*^9^. Other ffs consumed in SEA include palm-based products (Sri Lanka), rice/cassava-based (Philippines, Vietnam, Thailand, Indonesia, Malaysia), Soy-based (Thailand, Japan), milk-based (Thailand, Vietnam, Japan, China, Korea), and fish-based products (Thailand, Cambodia, Vietnam, Japan).

The presence of undesirable toxigenic compounds such as mycotoxins and plant toxins especially in amounts in ffs beyond the approved limits may cause serious health problems among consumers or even death. *Aspergillus* spp., *Penicillium* spp., *Mucor* spp., and *Rhizopus* spp. have been detected in grains, aflatoxins in commercial beverages, and ochratoxin A and zearalenone in some local beverages^10^. Plant toxins e.g., cyanogenic glycosides have also been reported in cassava and bamboo shoots^11^. In fermented milk products, the presence of *Streptococcus* spp. and *Clostridium botulinum* have led to severe outbreaks^12^. The quality of ffs becomes doubtful and influences the market especially if this problem occurs concurrently. Hence, there is a need for various precautions in food industries for ensuring high-quality products and safe maximum excess levels of pathogenic microorganisms and toxins in the food products^8^.

This review summarizes the most found toxins (mycotoxins and plant toxins) in traditional fermented foods in SEA, their sources, and mitigation measures for preventing health risks associated with their consumption.

## Fermented foods

As defined by Campbell-Platt^13^, ffs are foods that are produced by the action of microorganisms or enzymes so that beneficial biochemical changes can generate significant changes in the food. The fermentation process can take place either in a solid-state or liquid state (submerged fermentation). For the solid-state, aerobic microorganisms are responsible for the fermentation process, while the submerged fermentation takes place under anaerobic conditions^14^. The ffs can be grouped in various ways^15^; (1) by categories of fermentation process^4,16^—alcoholic fermentation, acetic acid fermentation, lactic acid fermentation, and alkaline fermentation; (2) by food commodity classes^13^—dairy-based products, cereal-based products, legume-based products, fish and seafood-based products, meat-based products, root, and tuber-based products and beverages; (3) based on types of microorganism involved^17^ - yeasts, molds, bacteria (acetic bacteria, lactic acid bacteria (LAB) and *Bacillus*).

### Traditional fermented foods in Southeast Asia

Fermented foods are associated with diverse cultures and civilizations. Climate, history, and the raw materials produced particularly in the regions have led to the exploitation of different fermentation mechanisms to produce indigenous edible foods and beverages which are adapted to the particular environmental conditions^18^. For instance, in locations where there are animal husbandry and pastoral agricultural practices, there is the general availability of milk and dairy products from cows, goats, sheep, etc. Accordingly, fermented milk, cheese, and other fermented dairy products have evolved across Europe, Middle East, and India. On the contrary, far Eastern regions including Japan, China, and Korea have limited animal husbandry^19^. Several factors such as economic factors, cultural and religious status also affect the categories of substrates used in producing ffs and alcoholic beverages. In Asia, ffs that have evolved over the years are generally based on rice (and grains), vegetables, soybeans, and fish as main substrates^18^. In recent years, ffs have received great attention from food industries, and their rising global market was estimated to be about thirty billion USD^18^. In SEA ffs are still often produced at a family- or community-scale using traditional techniques and as well on an industrial scale. Studies on indigenous ffs were searched for, and relevant works including those carried out by Tamang^20^; Liu and Tong^21^; Tamang, et al.^22^; Swain, et al.^23^; Beuchat^24^; Steinkraus^17^; Soni and Dey^25^; Owens^26^ were reviewed and presented in Table 1. From these papers, indigenous ffs and beverages peculiar to the region of SEA were extracted together with their local names, countries of origin, raw materials/substrates, and organoleptic properties. Thus, the indigenous ffs and beverages in SEA have been classified into eight different groups according to their food categories (Table 1a–h). These include fermented dairy products, fermented cereal products, fermented fruits and vegetables, fermented legume products, fermented root crops, fermented meat products, fermented fish products, and fermented alcoholic beverages.

**Table 1:** **a** Indigenous dairy products in SEA, **b** indigenous fermented cereal products in SEA, **c** indigenous fermented fruits and vegetable sin SEA, **d** indigenous legume products in SEA, **e** indigenous fermented root crop products in SEA, **f** indigenous fermented meat products in SEA, **g** indigenous fermented fish products in SEA, **h** indigenous fermented alcoholic beverages in SEA.

*Local Name*	*Country*	*Raw material*	*Organoleptic properties*
**a**. **Fermented dairy products**			
*Dadih*	Indonesia	Buffalo milk	Curd, savory
*Sua Chua*	Vietnam	Dried skim milk, starter, sugar	Acid fermented milk
**b. Fermented cereal products**			
*Ang-kak*	Thailand, Philippines	Red rice	Colorant
*Dosa*	Malaysia, Singapore	Rice and black gram	Thin crisp pancake, shallow fried
*Idli*	Malaysia, Singapore	Rice and black gram or other dehusked pulses	Soft moist, spongy, mild-acidic
*Khaomak (Kao-mak)*	Thailand	Glutinous rice *Look-pang* (starter)	Sweet and mild alcoholic Thai desert
*Puto*	Philippines	Rice	Steamed rice cake
*Tape Ketan*	Indonesia	Glutinous rice, *Ragi*	Sour, sweet, mild alcoholic desert
**c. Fermented fruits and vegetable products**			
*Burong mustala*	Philippines	Mustard	Wet and acidic
*Dha muoi*	Vietnam	Eggplant, mustard, and beet	Wet and acidic
*Hom-dong*	Thailand	Red onion	Fermented red onion
*Naw-mai-diong*	Thailand	Bamboo shoots	Wet and acidic
*Pak-gard-dong*	Thailand	Leafy vegetable, salt, and boiled rice	Wet, acidic, side dish
*Pak-sian-dong*	Thailand	*Gynandropis pentaphylla* leaves	Wet, acidic, side dish
*Dakguadong*	Thailand	Mustard leaf	Wet, acidic, sour, salad
*Sayur asin*	Indonesia	*Mustard leaves, coconut, salt, and cabbage*	Wet, acidic, sour, salad
*Tempoyak*	Malaysia	Durian (*Durio zibethinus*), salt	Wet, acidic, side dish
**d. Fermented legume products**			
*Kecap*	Indonesia	Wheat, soybean	Liquid
*Ketjap*	Indonesia	Black soybean	Syrup liquid
*Oncom Hitam (black) and Oncom Merah (orange)*	Indonesia	Tapioca peanut press cake, soybean cord starter	Fermented peanut press cake fried or roasted
	Indonesia		
*Tauco*	Indonesia	Soybean	Alkaline, liquid, seasoning
*Tempeh*	Indonesia	Soybean	Paste, flavoring agent, alkaline
*Thua nao*	Thailand	*Soybean*	Dry, paste, side dish, alkaline
**e. Fermented root crop products**			
*Tapé*	Indonesia	Cassava	Dessert (sweet)
*Tapai Ubi*	Malaysia	Cassava, *Ragi*	Dessert (sweet)
**f. Fermented meat products**			
*Hham (Musom)*	Thailand	Pork meat, pork skin, rice garlic, ginger, salt	Fermented pork
*Nem-chua*	Vietnam	Pork, cooked rice, salt	Fermented sausage
*Nham or Sai-krok-prieo*	Thailand	Pork, rice salt, garlic	Fermented sausage
*Tocino*	Philippines	Pork, potassium nitrate, salt, sugar	Fermented cured pork
**g. Fermented fish products**			
*Balao-balao*	Philippines	Shrimp, rice, salt	Fermented rice shrimp, condiment
*Belacan*	Malaysia	Shrimp salt	Condiment, paste
*Bakasang*	Indonesia	Shrimp, fish	Condiment, paste
*Burong Bangus*	Philippines	Milkfish, vinegar, rice, salt	Fermented milkfish, sauce
*Burong Isda*	Philippines	Fish, rice, salt	Fermented fish sauce
*Budu*	Malaysia, Thailand	Marine fishes, sugar, salt	Fish sauce, Muslim sauce
*Hoi-malaeng pu-dong*	Thailand	Mussel (*Mytilus smaragdinus*) salt	Fermented mussel
*Nam pla*	Thailand	*Solephorus sp*., *Ristelliger sp*. *Cirrhinus sp*., water, salt	Fish sauce
*Nuoc mam*	Vietnam	Marine fish	Fish sauce, condiment
*Patis*	Indonesia, Philippines	*Stolephorus* sp., *Clupea* sp., *Decapterus* sp., *Leionathus* sp., salt	Fish sauce
*Pla-paeng-daeng*	Thailand	Marine fish, *Ang-kak* (red molds rice), salt	Red fermented fish
*Pla-som (Pla-khao-sug)*	Thailand	Marine fish, boiled rice, salt, garlic	Fermented fish, condiment

*Beverage name*	*Country*	*Raw material*	*Organism/starter*	*Organoleptic properties*
**h**. **Fermented alcoholic beverages**				
*Basi*	Philippines	Sugar cane	*Bubod, binubudan*	Cloudy or clear liquid
*Brem*	Indonesia	Rice	*Ragi*	Mild alcoholic, sweet-sour, dry
*Khao maak*	Thailand	Rice	*Lookpang*	Sweet taste, white colored, juicy, mild alcoholic
*Krachae*	Thailand	Rice	*Lookpang*	Filtered liquor non-distilled
*Nam khao*	Thailand	Rice	*Lookpang*	Distilled liquor
*Ou*	Thailand	Rice	*Lookpang*	Distilled liquor
*Sato*	Thailand	Rice	*Lookpang*	Distilled liquor
*Ruou de, Ruou nep*	Vietnam	Rice	*Men*	Clear, distilled liquor
*Ruou nep than*	Vietnam	Purple rice	*Men*	Viscous thick, non-distilled fermented rice
*Ruou nep chan*	Vietnam	Maize, rice, cassava	*Men*	Viscous, thick, distilled/non-distilled fermented rice
*Tapuy*	Philippines	Rice	*Bubod*	Sour, sweet, mild alcoholic
*Tapai ubi, Tapai pulut*	Malaysia	Cassava, rice	*Ragi/ jui-piang*	Sour, sweet, mild alcoholic
*Tapé-ketan*	Indonesia	Cassava, rice, millet, maize	*Ragi*	Sweet-sour alcoholic paste

Fermented dairy products constitute about 20% of the entire economic value of ffs produced globally. Strains of LAB such as *Lactobacillus, Lactococcus*, and *Leuconostoc* are generally employed in the fermentation of dairy products^27^. Yogurt is well known as the major fermented milk worldwide and is prepared mainly from the fermentation of cow milk by two species of LAB (*Streptococcus thermophilus* and *Lactobacillus delbrueckii* subsp. *Bulgaricus*)^2^. Similarly, cheese and other products are made from non-pasteurized milk and may still depend on lactic microbes for fermentation, especially on an industrial scale. Indonesian *Dadih*, produced from the fermentation of buffalo milk and Vietnamese *Sua Chua* from dried skimmed milk are examples of common fermented milk found in SEA.

Fermented cereal products play a major role in human nutrition in all regions of the world, including Asia. Among all ffs, fermentation of cereals is the most fermented product, attaining the highest volume^28^. Generally, cereal-based ffs are majorly produced from rice, maize, sorghum, millet, or wheat. However, most fermented cereal-based products in SEA are mainly derived from rice, which is either liquid (porridge) or stiff gels (solid) in texture. These include *Dosa* (Malaysia, Singapore), *Idli* (Malaysia, Singapore), *Khaomak* (Thailand), *Puto* (Philippines), and *Tapé Ketan* (Indonesia).

Fermentation is one of the oldest processing methods for extending the shelf-life quality of perishable foods like fruits and vegetables, especially before storage in the refrigerator. They are various final products of fermented fruits and vegetables produced in SEA based on the kind of substrates used. Most fermented fruits and vegetables in SEA are indigenous to Thailand, the Philippines, Vietnam, Indonesia, and Malaysia. Mustard, beets, eggplant, red onion, bamboo shoots, and durian are mostly fermented into wet and acidic products which are often eaten as side dishes or salads.

Soybean is the major fermented leguminous crop fermented in SEA and products include *Kecap, Ketjap, Oncom Hitam* (*black*), *Oncom Merah* (*orange*)*, Tauco, Tempe*, and *Thua-nao* which are mainly produced in Indonesia and Thailand. These fermented products are in the form of syrup liquid, paste, or press cake used as a flavoring agent, seasoning, and side dish.

Processing and consumption of fresh, cured, and fermented meat products have been in practice in the world for many years. Fermented meats such as sausage-like products are not uncommon in European countries and Asia. In *Hham (Musom)*, *Nem-chua, Sai-krok-prieo*, and *Tocino* produced basically from pork meat are mostly found in Thailand, Vietnam, and the Philippines, respectively.

The origin of fermented fish products in SEA dates back to around 200 BCE to 200 CE and Thai-Lao, Burmese, and Khmer are concluded as the first ethnic groups to produce fermentation of freshwater fish products^20^. Marine fishes, shrimps, and mussels are generally fermented into fermented shrimp paste, fermented fish sauce, condiments as shown in Table 2.

**Table 2:** Sample preparation and analytical techniques for the determination of mycotoxins in SEA fermented foods and raw materials used for preparing fermented foods.

Commodity	Country	No of samples	Extraction Solvent	Clean -Up	Analytical method	Mycotoxins	Reference
Fermented Wine and Beer	Malaysia	10	Acetonitrile	SPE	LC-MS/MS	AF, OTA	Alsharif, et al.^140^
	Thailand	200	Chloroform/Acetonitrile	DLME	LC-MS/MS	AF, ZEN, OTA, FB, T-2, DON	Puangkham, et al.^141^
Dried Chilli	Thailand	120	Methanol	*	TLC	AF, OTA	Chuaysrinule, et al.^142^
	Indonesia	6	Methanol-Water	IAC	HPLC-UV	AF, OTA	Wikandari, et al.^143^
	Malaysia	10	Acetonitrile	SPE	LC-MS/MS	AF, OTA	Alsharif, et al.^140^
	Malaysia	80	Methanol-NaHCO_3_	IAC	HPLC-FL	AF, OTA	Jalili and Jinap^144^
Rice	Thailand	10	Methanol	SPE	LC-QTOFMS	AF, ZEN, OTA, FB, T-2, DON	Shiratori, et al.^145^
	Vietnam	111	Methanol-Water	*	ELISA	AF, FB	Huong, et al.^146^
	Thailand	240	Methanol	SPE	HPLC-FL	AF, FB	Panrapee, et al.^147^
	Myanmar	21	Acetonitrile	SPE	LC-MS/MS	AF, FB	Lim, et al.^148^
	Malaysia	50	Methanol	IAC	ELISA	AF, OTA	Lim, et al.^148^
Fish	Philippines	31	Ethanol	SPE	HPLC-UV	AF, OTA	Ebarvia, et al.^149^
Soybean (Tempeh)	Indonesia	9	Acetonitrile-Water	DnS	HPLC-MS/MS	ZEN	Borzekowski, et al.^150^
Palm Kernel Cake	Indonesia	20	Methanol-water	*	ELISA	AF	Reiter, et al.^151^
	Malaysia	25	Acetonitrile-Water	*	LC-MS/MS	AF, ZEN, OTA, FB, T-2, DON	Yibadatihan, et al.^152^
Corn and Cornmeal	Vietnam	102	Methanol-Water	*	ELISA	AF, FB	Huong, et al.^146^
	Thailand	55	Methanol		HPLC-UV	OTA	Singkong, et al.^153^
	Indonesia	13	Methanol	BSA	ELISA	AF	Reiter, et al.^151^
	Indonesia	16	Acetonitrile	IAC	HPLC-UV	AF, ZEN, OTA, FB, T-2, DON	Ali^154^
	Philippines	50	Acetonitrile-Water	SPE	HPLC-FL	AF, ZEN, FB	Yamashita, et al.^155^
	Thailand	27	Acetonitrile-Water	SPE	HPLC-FL	AF, ZEN, FB	Yamashita, et al.^155^
	Indonesia	12	Acetonitrile-Water	SPE	HPLC-FL	AF, FB	Yamashita, et al.^155^

*AF* aflatoxins, *ZEN* zearalenone, *OTA* ochratoxin, *FB* fumonisins, *T-2* T-2 toxin, *DON* deoxynivalenol, *ELISA* enzyme linked enzyme linked immunosorbent assay, *TLC* thin layer chromatography, *HPLC* high performance liquid chromatography, *FL* fluorescence, *UV* ultraviolet, *MS* mass spectrometry, *SPE* solid phase extraction, *DnS* dilute and shoot, *IAC* immunoaffinity column, *BSA* bovine serum albumin, *ND* not detected.

*Not stated or investigated.

Cassava roots are also fermented into popular Indonesian and Malaysian sweet deserts known as *Tapé* and *Tapai Ubi*, respectively. Ragi, a dry starter culture consisting of bacteria, yeast, and mold is generally used in this process.

Sugarcane, rice, maize, millet, and cassava serve as common substrates for alcoholic beverages in SEA using *Bubod, binubudan, Ragi, Loogpang, Men* as starters. Popular alcoholic beverages in this region include Thai *Sato* produced from rice, Vietnamese *Ruou de, Ruou nep, Ruou nep than, Ruou nep chan* from rice/purple rice, maize, and cassava, and Indonesian *Tapé-ketan* from fermented rice, cassava, maize, and millet.

## Contamination sources in fermented foods

Contamination in fermented foods can occur either during the primary production of plant/animal-based raw materials or during fermentation itself. Contamination during or after fermentation can also be due to inadequate hygienic conditions or appropriate packaging systems. Several raw materials used in food fermentation naturally contain toxic compounds, e.g., cyanogenic glycosides in cassava, while mycotoxin contamination may take place during pre-and post-harvest of the plant-based raw materials. In addition, toxins such as biogenic amines (Histamine, putrescine, tyramine, cadaverine, and *β*-phenylethylamine, ethyl carbonate), ethyl carbonate, and bacterial toxins may be released as by-products into the ffs during fermentation.

The fact that ffs are still generally produced at community-scale or family-scale using traditional methods reduces the consumer confidence in the quality and safety of the products, and it is highly important to improve the method of production. For example, consumers do not trust the microbial safety of *Nem-chua* (Vietnamese fermented sausage) because of the lack of confidence in the microbial safety of the traditionally produced meat^29^.

### Mycotoxin contamination in Southeast Asian fermented foods

All fungal toxins are referred to as mycotoxins which are mainly produced by five species of filamentous fungi, namely *Fusarium, Aspergillus, Penicillium, and Alternaria*. Recently, more than 400 mycotoxin metabolites have been identified, however, the eight most significant mycotoxin with global relevance regarding public health are aflatoxins, ochratoxin A, zearalenone, deoxynivalenol, fumonisin B_1_, nivalenol, T-2 toxin and patulin^30^. Mycotoxin contamination has been discovered in agricultural commodities and specifically in cereals and legumes including rice, wheat, maize, barley, oats, rye, beans, soybeans, peanuts, clover, peas, alfalfa, lentils, chicken peas, and in some other grains that are generally used as raw materials for traditional ffs in SEA^4^.

**Fig. 1:**
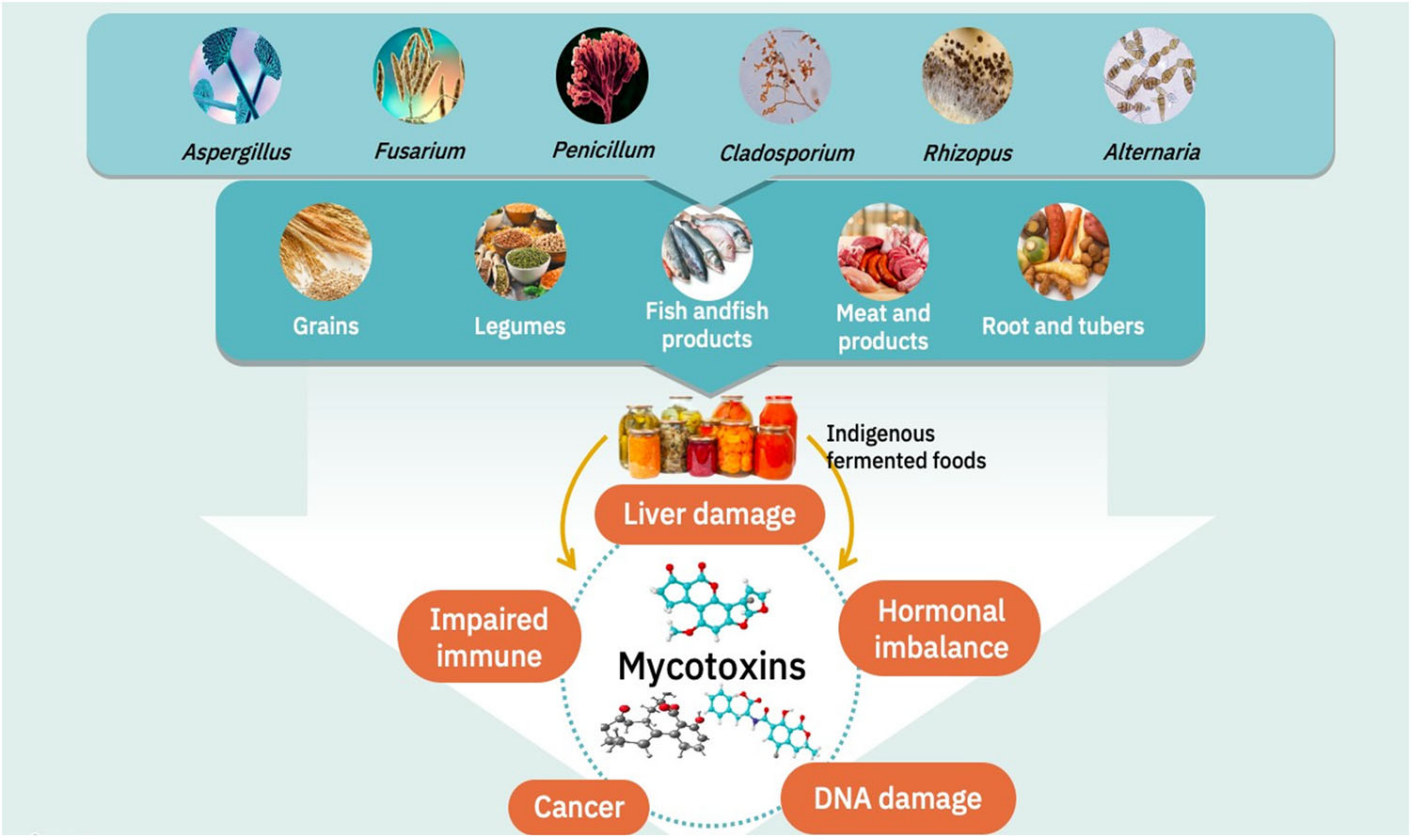
Mycotoxin contamination in indigenous fermented foods and health implications on human. Illustrations adapted from Adobe stock images (http://stock.adobe.com/) under Standard Licensing.

In SEA, diverse mycotoxins including aflatoxins, ochratoxin A, zearalenone, deoxynivalenol, and citrinin have been recorded in ffs (Fig. 1). Studies carried out by Inoue, et al.^31^ on maize, barley, and other grains used to produce beer traditional and commercial alcoholic beverages, were assessed for mycotoxins. They observed that the grains were contaminated with *Aspergillus* spp., *Penicillium* spp., *Mucor* spp., and *Rhizopus* spp., and some of the commercial beverages were contaminated with AFs, while a few of the local beverages contained zearalenone and ochratoxin A.

In Asian ffs, *Rhizopus* spp. is generally used in the fermentation process. Some of these fungi species have over time become opportunistic pathogens for consumers with the impaired immune system, while some others have an endosymbiotic interaction with *Burkholderia*, which has the potential of producing toxic metabolites. Investigation into several strains of *Rhizopus* showed that about 11% of the strains possess an endosymbiotic association with *Burkholderia* spp.^32^. The *Burkholderia* strains were studied and proven to exhibit the potential of producing rhizoxins.

**Fig. 2:**
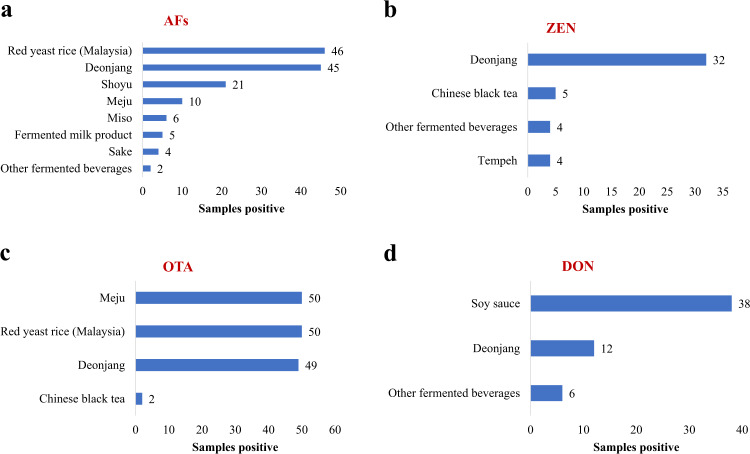
Incidence of mycotoxin contamination in Asian fermented foods. **a** Frequency of AFs contamination in Asian fermented foods samples. **b** Frequency of ZEN contamination in *n* Asian fermented foods samples. **c** Frequency of OTA contamination in Asian fermented foods samples. **d** Frequency of DON contamination in Asian fermented foods samples. AFs Aflatoxins, ZEN Zearalenone, OTA Ochratoxin A, DON Deoxynivalenol.

**Fig. 3:**
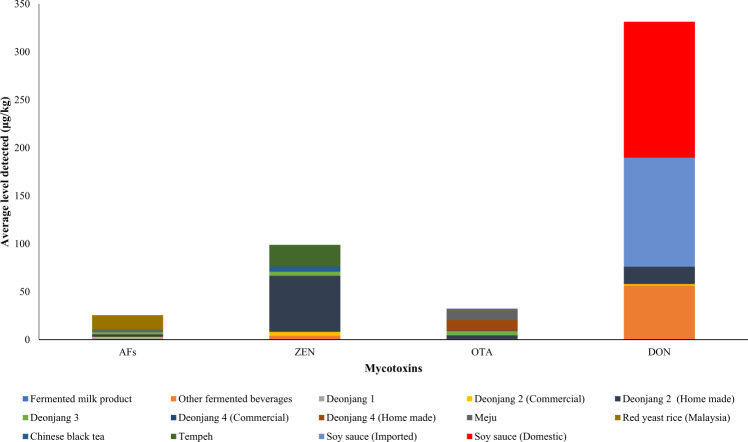
Maximum mycotoxin concentrations detected in Asian ffs. AFs Aflatoxins, ZEN Zearalenone, OTA Ochratoxin A, DON Deoxynivalenol. *Deonjang 1–4 indicated samples analyzed by different authors.

Notwithstanding limited information on the mycotoxin contamination in Southeast Asian ffs, publications relevant to this issue were sourced from the published literature and analyzed. Incidence of mycotoxin contamination in ffs were extracted from fifteen publications and summarized in Fig. 2. The ffs included those from fermented soybean (*Deonjang, Meju, Miso, Shoyu, Soy sauce, tempeh*); rice (red yeast rice (RYR) and sake); fermented milk products and alcoholic beverages. Most of the AF contaminations were recorded in RYR from Malaysia (*n* = 46/50)^33,34^ and *Deonjang* (*n* = 45/118)^35–38^. Zearalenone mostly occurred in *Deonjang* samples (*n* = 32/60)^35^; ochratoxin A mainly in Meju (*n* = 50/100)^37^, RYR from Malaysia (*n* = 50/100)^33^ and *Deonjang* (49/109)^33–35^; deoxynivalenol in Soy sauce (*n* = 38/40)^35^ and *Deonjang* (*n* = 12/60)^35^. RYR were the most vulnerable to citrinin contamination as the number of samples from Malaysia and China positive to citrinin were *n* = 50/50 and *n* = 43/114, respectively^33,34,39^. Lastly, fumonisin contamination only occurred in *Deonjang*, where 30 of 60 samples evaluated were found positive to fumonisin^39^. Figure 3 shows the maximum mycotoxin concentrations detected in the ffs. RYR from Malaysia was found to contain aflatoxins in average concentrations (2–15 µg/kg of aflatoxins) exceeding the maximum levels (4 µg/kg of aflatoxins) by The European Union Commission^40^. Also, highest average concentration of zearalenone (58 µg/kg of zearalenone), was detected in homemade *Deonjang*, which is quite below the maximum level (75 µg/kg of zearalenone) by The European Union Commission^41^. Average concentrations of samples found to contain ochratoxin A ranged between 1–11 µg/kg of ochratoxin A, with all higher than the regulated limit (3 µg/kg of ochratoxin)^42^, except for RYR and commercial *Doenjang* which only contained 1 µg/kg of ochratoxin A. Average deoxynivalenol concentrations detected in both imported and domestic soy sauce were found to be 114 µg/kg and 142 µg/kg of deoxynivalenol, respectively, below the regulated limit (750 µg/kg of deoxynivalenol)^41^. The average citrinin concentration in RYR from China were found to be 884 mg/kg of citrinin, which may be of serious safety concerns in this ff product when compared to the maximum limit (100 µg/kg of citrinin)^43^. From this study, a total of 1492 ffs samples consumed in Asia including SEA were evaluated for six different types of mycotoxins and 514 samples (26%) were recorded to be contaminated with at least one type of mycotoxin. Ochratoxin A and aflatoxins were the major mycotoxins identified, representing 29% and 27%, respectively of the total contamination.

Mycotoxins have also been detected in Thai fermented soy products (*Thua-nao*) and *Tuong* as reported in works by^44^ and^45^. *Tuong* is fermented by *Aspergillus oryzae*, a domesticated type of *Aspergillus flavus* (AF producer), evidence that explains the presence of AF in brands of *Tuong*. Aflatoxins are predominantly found in fermented milk products. Different factors influence the free aflatoxins in fermented milk products including storage temperature, storage time, pH, acidity, aflatoxin concentration, and strains used in the fermentation process^46^. Similarly, aflatoxins have been reported in peanut/soybean press cakes such as Indonesian *Oncom Hitam* and *Oncom Merah* that are produced using *Neurospora sitophila* and *Rhizopus oligosporus*^47^. *Rhizopus oligosporus* can reduce the cyclopentanone moiety resulting in aflatoxicol A, which is about eighteen times less toxic than aflatoxin B_1_. In all, there is still a major research gap on mycotoxin contaminations, especially in SEA indigenous ffs as there are not much data on this subject.

### Phytotoxin contamination in Southeast Asian fermented foods

#### Cyanogenic glycosides

**Fig. 4:**
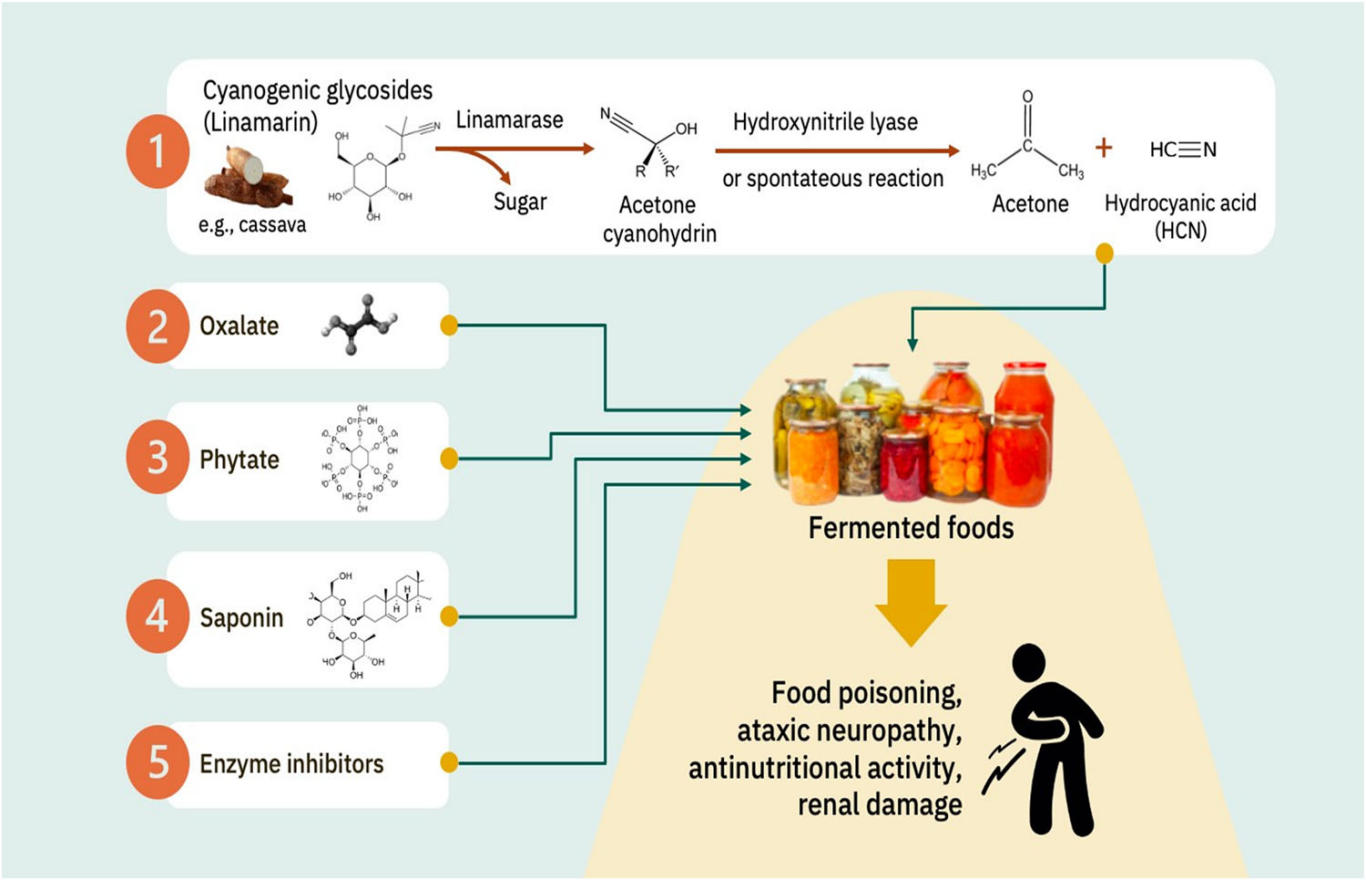
Plant toxins from substrates to fermented foods. Illustrations adapted from Adobe stock images (http://stock.adobe.com/) under Standard Licensing.

Enzymatic or acid hydrolysis of cyanogenic glycosides produces hydrocyanic acid (HCN), which is strong enough to suppress respiration^48^. Hydrocyanic acid suppresses cytochrome oxidase, and the enzymatic disintegration of cyanogenic glycosides often takes place in two phases (Fig. 4). In the first phase, beta-glycosidase hydrolyzes the beta-glycosidic bond present between the sugar and aglycone, also known as cyanohydrin. Lyases including hydroxy nitrile lyases catalyze the decomposition of cyanohydrin to HCN and a ketone or an aldehyde, according to the kind of glycoside in the second stage. Cyanogenic glycosides are generally present in tubers of cassava and beans. Cassava tubers consist of two types of cyanogenic glycosides, namely linamarin (95%) and lotaustralin. Cassava tubers contain linamarase (beta-glucosidase), which encounters linamarin (substrate) during the disruption of the cellular structure of the tuber (e.g., grating or grinding process). This may consequently lead to the enzymatic hydrolysis of cyanogenic glycosides and the release of HCN together with unhydrolyzed glycosides. Most human poisonings by cyanogenic glycosides in the tropics including SEA are mainly a result of consumption of cassava products as they still exhibit significant concentrations of HCN regardless of the processing^49^. Hydrocyanic acid has been reported in tropical ataxic neuropathy, a category of nervous disorders. However, traditional methods of producing cassava-based ffs such as fermentation, soaking, and pressing of soaked pulp have proven effective in eliminating some amounts of HCN.

### Oxalates

Oxalates are scarcely absorbed under non-fasting circumstances in humans. In situations where foods containing excess amounts of oxalates are consumed, they may be excreted as insoluble calcium salts^50^. There is little knowledge on the oxalate contents present in cereals, legumes, and their products. However, the process of fermentation removes up to 43% of oxalates in some fermented legumes cereals such as soybeans and peanuts and this could be further reduced if subjected to soaking, dehulling, and cooking^47^. Excess ingestion of oxalates especially from fermented plant foods may inhibit calcium metabolism, resulting in chronic illnesses including renal damage and stone formation.

### Phytates

Phytate (myoinositol 1,2,3,4,5,6hexakis dihydrogen phosphate) exists mainly as a result of the monovalent and divalent cations in distinct regions of cereal grains, legumes, and few roots and tubers^48,51^, where it comprises 85% of the total phosphorus. The presence of phytic acid in ffs causes serious concern as it reduces mineral bioavailability as well as their solubility, functionality, and protein digestibility by forming complexes. This antinutritional substance may also interact with enzymes including alpha-amylase, pepsin, trypsin, and beta-glucosidase, leading to a reduction in their activities. There are two recognized kinds of phytases, namely 3-phytase and 6-phytase. The 6-phytase is commonly found in deeds and grains of higher plants, while the 3-phytase seems to have attributes of microorganisms. During some fermentation and other food processes of tubers, cereals, and grains, phytase contents may be reduced to a certain extent. Phytic acid hydrolysis in *Tempeh* during fermentation by the action of phytase, which is released during fermentation by *Rhizopus oligosporus*, thereby reducing the phytate contents in the ffs. Phytic acid contents have been observed to reduce up to 54.5% in *Tempeh* after fermentation, though it calls for more strategies for safety evaluation^52^.

### Saponins

Saponins are regarded as surface-active sterol or triterpene glycosides to which pentoses or hexoses are linked to sapogenin, a non-polar group that could either be a steroid or a triterpene^53^. The presence of saponins in foods can easily be identified through their hemolytic activity and their capacity of forming stable foams in aqueous solutions. Although saponins seem to be practically non-toxic to humans, they have been studied to low cause cell inhibition^54^. Saponins cannot be destroyed by cooking, however, fermentation may reduce the amounts in foods. Studies on decontamination by fermentation carried out by Fenwick and Oakenfull^55^; Reddy et al.^51^ revealed a 55.8% decrease in the saponin contents of soybean *Tempeh* after fermenting with *Rhizopus oligosporus*.

### Enzyme inhibitors

Some naturally existing compounds in plants can inhibit proteolytic and amylolytic enzymes including trypsin, amylase, and chymotrypsin. Trypsin inhibitors can suppress growth by interfering with the digestion of protein, excessive production of pancreatic enzymes, pancreatic hypertrophy, and metabolic disruption in the use of sulfur amino acids in animals fed with plant products containing high concentrations of trypsin inhibitors^56^. In fermented plant foods, alpha-amylase inhibitor activity has scarcely been reported. The process of fermentation with microorganisms, cooking and controlled heat treatments may reduce the number of enzyme inhibitors in the substrates used in producing ffs. Tannins and some polyphenols are examples of plant-based enzyme inhibitors that form complexes with proteins, leading to the inactivity of the enzyme, decrease in protein solubility and digestibility and reduced ions absorbed^57^. Enzymes inhibited by these compounds include trypsin, pepsin, chymotrypsin, glucosidase, amylase, and lipases. Fermentation with appropriate fungi or bacteria can help achieve optimal pH conditions for the enzymatic hydrolysis/degradation of tannins and polyphenols^58^. Fermentation of cooked common beans with *Rhizopus oligosporus* (*Tempe* fungi) resulted in a 47% decrease in the activity of trypsin inhibitor due to hydrolysis by the action of *Tempe* fungi^59^.

### Biogenic amines

Biogenic amines (BA) are low-molecular-weight nitrogenous compounds formed in biological systems by enzymatic decarboxylation of certain amino acids or by amination and transamination of aldehydes and ketones^60^. BAs are mainly produced in food and beverages by thermal or bacterial decarboxylation of free amino acids during preservative technological processes such as pasteurization and fermentation^61^. Lactic acid bacteria and some bacteria belonging to family *Enterobacteriaceae* are the main producers of BA in ffs^62,63^. Proteolysis and acidification during fermentation process do not only allow these BA-producers to persist and thrive, but also increase the availability of precursor amino acids, resulting in accumulation of BAs^63,64^.

The most common BAs found in food are histamine (HIS), tryptamine (TRY), putrescine (PUT), spermine (SPM), cadaverine (CAD), agmatine (AGM), spermidine (SPD), *β*-phenylethylamine (PHE), tyramine (TYM) and trimethylamine (TMA)^60,65^. Low levels of these compounds in food are not considered as a serious risk. However, when present in high concentrations, they could induce wide range of adverse health effects in consumers, especially in sensitive individuals^66–68^. HIS and TYR are the most widely studied BA due to their frequent occurrence in ffs and toxic effects including headache, hypotension, and gastroenteritis^67^. Although, the toxicity of other amines such as PUT, CAD, TRY and PHE remain elusive. However, they have been found to exacerbate the negative effects of HIS and TYR, due to inhibition of HIS and TYR metabolizing enzymes^66^. Moreover, they can react with nitrites in food, to produce carcinogenic nitrosamines^66,68,69^. In terms of regulation, HIS is the only BA with regulatory limits. The US Food and Drug Administration (USFDA) set a maximum limit (ML) of 50 mg/kg, while the European Food Safety Authority (EFSA) established a ML of 100 mg/kg for HIS in fish and fish products intended for human consumption^65^.

It is noteworthy to state here that other plant toxins such as pyrazolidine and tropane alkaloids have been identified in certain cereal crops due to the weeds that grow among the cereals. These toxins may also contribute to toxin contaminations in ffs, although it is an uninvestigated risk.

## Analytical techiniques for determination of common natural toxins in sea fermented foods

### Mycotoxins

Accurate determination of mycotoxins in ffs can be challenging due to the structure and chemical composition of the matrices. Thus, most sample preparation for mycotoxin analysis involves an extraction and clean-up steps prior to instrumental analysis. Due to the vast variety of raw materials that are fermented, diverse extraction and clean-up procedures can be found in the literature (Table 2)^70,71^. Also, the choice of solvents has been shown to contribute significantly to the maximal recovery of analytes of interest. Generally, mixtures of methanol–water and acetonitrile-water are the most frequently used extraction solvents during sample preparation^72–74^. However, for ffs with high fat content and pigments, extraction solvents including ethyl acetate–formic acid, 1-octanol and toluene, dichloromethane, acetone, and chloroform have been found to be more efficient for the recovery of mycotoxins^75^. The clean-up step following sample extraction helps to improve selectivity and reduce interference compounds (matrix effect), which in turn, contribute to the accurate measurement of mycotoxins in ffs. Clean-up methods commonly employed for food analysis include solid phase extraction (SPE), liquid–liquid extraction (LLE), solid–liquid extraction (SLE), accelerated solvent extraction (ASE), supercritical fluid extraction (SFE), microwave-assisted extraction (MAE), vortex assisted low density solvent–microextraction (VALDS–ME), solid phase extraction (SPE), bovine serum albumins (BSA), aptamer-affinity columns (AACs), molecularly imprinted polymers (MIPs) and immunoaffinity columns (IAC)^73–75^. The most frequently used clean-up methods for the determination of mycotoxins in ffs are SPE, IAC and BSA.

The SPE technique is fast, requires less solvent, and remove interfering substances present in the food matrix by sorption on a solid adsorbent (usually packed in cartridges), while producing a solution containing mainly the analyte of interest—mycotoxins^76^. Examples of sorbents used for sample clean-up include C-18 and primary and secondary amine (PSA). In contrast to SPE, IAC isolate and purify mycotoxins through selective binding of antibodies immobilized onto small columns. Analytes are eluted from the column using minimal amounts of organic solvent^77^. IAC have been developed for both single and multi-mycotoxin, especially the regulated mycotoxins^77,78^. Despite providing reliable results, IAC still have some important drawbacks such as matrix interference, cross reactivity, column’s short life, column capacity, and are very expensive^77^. Serum albumins from bovine origin (BSA) that can mimic the recognition properties of antibodies and form a stable binding affinity towards some mycotoxins are currently being used to develop BSA-based sample clean-up columns. They are cost-effective, precise, and accurate as IAC^70,79^.

Several analytical techniques including immmunoassays, TLC, gas chromatography, and high-performance liquid chromatography coupled with various detectors like FL, diode array, UV, and MS, have been developed and used for the detection and quantification of mycotoxins in ffs (Table 2)^80^. The most used techniques for determination of mycotoxins in SEA ffs are HPLC-UV, HPLC-FL, and LC-MS/MS (Table 2). For precise and accurate results, and simultaneous determination of multiple mycotoxins at low quantification limits, LC-MS/MS is the method of choice. However, when rapid analysis of mycotoxins is required, immunoassay-based methods including enzyme-linked immunosorbent assay (ELISA), lateral-flow devices (LFDs) and biosensors are very useful despite their numerous drawbacks (such as false-positive results, sensitivity, and reproducibility).

### Biogenic amines

Several analytical methods have been developed to detect and quantify BA levels in ffs. These techniques range from the more traditional colorimetric and fluorometric methods; to chromatography methods including gas chromatography (GC) and high-performance liquid chromatography (HPLC) coupled with various detectors such as fluorescence (FL), ultraviolet (UV), photo-diode array (PDA) and mass spectrometry (MS)^81,82^. HPLC-UV and HPLC-FL are the most frequently reported methods for the detection and quantification of BA in SEA ffs (Table 3). Prior to instrumental analysis of samples, a pre-and post-column derivatization is required to accurately quantify BA. This is due to the lack of chromophore or fluorophore in BA structure^83,84^. Derivatization agents including dansyl chloride, fluorescamine, fluorenyl-methyl chloroformate, 6-aminoquinolyl-N-hydroxy-succinimidyl carbamate and O-phthaladehydes are suitable agents. However, dansyl chloride is the most preferred, as it can react with both the primary and secondary amino groups to form a more stable and highly sensitive derivatized products^84,85^.

**Table 3:** Sample preparation and analytical techniques for determination of biogenic amines in SEA fermented foods and raw materials used for preparing fermented foods.

Fermented Food	Local name	Country/Origin	No of samples	Biogenic amines	Extraction solution	Derivatisation agent	Analytical method	Reference
Wine	Mijiu, Uangjiu, Huangjiu	Malaysia	18	TRY, HIS, PUT, TYM, CAD, PHE, SPD	0.1 M HCl	Dansyl chloride	HPLC-PDA	Yue, et al.^156^
Fish	Sambal-terasi	Indonesia	6	TRY, HIS, PUT, TYM, CAD, PHE	5% TCA	Dansyl chloride	HPLC-UV	Damanik, et al.^157^
Fish and vegetables	Teuktrey, Prahok, Trasork chav	Cambodia	57	HIS, TYR, PUT, and CAD	0.4 M PCA	Dansyl chloride	HPLC-UV	Ly, et al.^158^
Pork	Nham	Thailand	4	TRY, HIS, PUT, TYM, CAD, PHE, SPD	0.4 M PCA	Dansyl chloride	HPLC-PDA	Santiyanont, et al.^83^
Shrimp paste	Kapi	Philippines	10	HIS, PUT, CAD, SPD, SPM	0.4 M PCA	Dansyl chloride	HPLC-UV	Pilapil, et al.^159^
Fish	Ikan pekasam	Malaysia	15	TRY, HIS, PUT, TYM, CAD, PHE, SPD, SPM	0.05 M HCl	Benzyl chloride	HPLC-UV	Ezzat, et al.^160^
Fish and vegetables	—	Malaysia	67	TRY, HIS, PUT, TYM, CAD, PHE, SPD, SPM	6% TCA	Benzyl chloride	HPLC-UV	Zare, et al.^161^
Cheese	—	Philippines	10	CAD, HIS, TYM	5% TCA	—	TLC	Vallejos, et al.^162^
Fish, soybean, and vegetables	Teuktrey, Prahok, Trasork chav, Tempe	Malaysia	62	TRY, PUT, HIS, TYM, SPD	5% TCA	Dansyl chloride	HPLC-UV	Saaid, et al.^163^
Bonito flakes	Katsuobushi	Philippines	8	TRY, HIS, PUT, TYM, CAD, PHE, SPD, SPM	5% TCA	Dansyl chloride	HPLC-UV	Qiao, et al.^164^
Soybean	Tempe	Indonesia	10	TYM, CAD, PHE, SPD, SPM	5% TCA	Benzyl chloride	HPLC-PDA	Nout, et al.^165^
Minced fish	Som-fug	Thailand	7	TRY, HIS, PUT, TYM, CAD, PHE	10% TCA	Dansyl chloride	HPLC-UV	Riebroy, et al.^166^

*LOD* limit of detection, *PCA* Perchloric acid, *TCA* Trichloroacetic acid, *HCl* Hydrochloric acid, *HPLC* High performance liquid chromatography, *TLC* Thin layer chromatography, *UV* Ultraviolet, *PDA* Photodiode-Array, *HIS* histamine, *TRY* Tryptamine, *PUT* putrescine, *SPM* spermine, *CAD* cadaverine, *SPD* spermidine, *PHE* β-phenylethylamine, *TYM* tyramine.

Recent advances in analytical techniques have paved the way for the use of biosensors and flow-injection analysis (FIA), which offer more advantages in comparison to chromatography techniques^86,87^. They are less-time consuming, much more economical, and do not require sample clean-up or derivatization procedures. Immobilized enzymes (such as diamine oxidase, amine oxidase, peroxidase, and histaminase) that are highly specific for the targeted BA are placed directly on the surface of a working electrode (biosensor) or in a suitable reactor in a FIA arrangement^88,89^. Most of the available systems also contain a second enzyme such as horseradish peroxidase, for direct detection of BA through chemiluminescence^88,89^.

In terms of incidences of BA in SEA traditional ffs, various studies have reported the BA content of fermented products as outlined in Table 3. PUT, CAD, TYR, and HIS were the most prevalent BA found in food, particularly in fish and vegetables from Cambodia and Malaysia (Table 3). HIS was detected mostly at levels exceeding the FDA and EFSA maximum permitted limits. The other amines (SPM, PHE and TRY) were detected at low concentrations, with mean total BA levels below 50 mg/kg. The range of total BA found in different types of fermented food as well as the country and analytical methods are summarized in Table 3.

## Mitigation strategies

This section discusses the various strategies that have proven effective in the removal or decontamination of mycotoxins and plants in ffs to ensure their safety.

### ‘Clean substrate’ strategy

Prevention of mycotoxin contamination in raw materials (cereal grains, legumes, fruits and vegetables, roots, and tubers, etc.) (Fig. 3) used in the production of indigenous ffs is of huge importance. The decontamination and detoxification of mycotoxins in agricultural commodities is a problem globally^90^. It is well known that the drying of crops immediately after harvesting (moisture contents < 14%), the proper handling, and proper storage conditions will aid in the in prevention of mycotoxin accumulation^91^. Physical processing such as screening and elimination of contaminated material and the peeling, grading, irradiation, segregation, washing, drying, and milling are common treatments applied to decontaminate^92^. Controlled storage environments such as appropriate ventilation, humidity, managed temperature, and packaging techniques curtail the growth of fungi and accumulation of mycotoxins.^93^. Essential oils including turmeric oil, clove oil, peppermint and lime oil (single-oil dose and when combined) has been shown to suppress both the production of aflatoxin B_1_ and the growth of *Aspergillus* species^94,95^. In rice grains, the use of the whole clove also inhibited the growth of *Aspergillus flavus* and *Penicillium citrinum* and thus reduced mycotoxins levels^96^.

### Fermentation process

The process of fermentation is one of the most economical and easiest ways of preserving foods besides adding organoleptic properties, nutritional quality, and health benefits to the ffs. Thus, fermentation helps in shelf-life extension of the ff product, facilitates transportation by reducing its volume, and eliminates undesirable elements^97^. In recent years, various microorganisms such as yeasts and molds, bacteria, have shown their ability to reduce or even eliminate mycotoxins^98^. Many researchers have reported fermentation process as an effective, sustainable, and inexpensive approach to reduce mycotoxins in food^99,100^. For instance, certain microbes in starter culture can adsorb mycotoxins to their cell wall or use them up as primary source of carbon and nitrogen^99,101^. Moreover, some enzymes produced by microorganisms have been shown to bio-transform or degrade mycotoxins, leading to mycotoxin reduction^44,99^. However, following food fermentation, ffs can still contain significant concentration of mycotoxins, particularly when the raw material or substrate is highly contaminated. Moreover, some fermentation processes provide favorable conditions for the growth and production of mycotoxins by toxigenic fungal species of *Aspergillus*, *Fusarium* and *Penicillium* genera^102,103^. In addition, fermentation conditions such as low pH, facilitate the hydrolysis of conjugated mycotoxins (such as deoxynivalenol-3-glucoside and zearalenone-16-glucoside) back to their precursor mycotoxins, which ultimately, result in the accumulation of mycotoxins. Therefore, there is a need for constant monitoring of mycotoxins in ffs and raw materials used for fermentation, to develop an accurate risk assessment and mitigation measures. More investigations on reducing plant toxins in ffs, specifically by fermentation needs to be carried out. In studies carried out by Shukla, et al.^46^, plants extracts (*Ginkgo biloba*, cloves, and *Nelumbo nucifera*), decreased fungal microflora when added during the fermentation of *Meju*. Aflatoxins are produced by the fungal isolates in *Meju*, while the addition of the plant extracts during the fermentation process enhanced the shelf-life quality and lowered the toxic effects from the fermented product. Conventional processes such as washing, decantation, milling, sieving, and fermentation of indigenous ffs have reduced mycotoxin levels by up to 90%^104^. Some fungi species are also effective in preventing mycotoxin contamination in ffs. For instance, *Aspergillus niger*, a strain of *A. flavus* that does not produce aflatoxins, when introduced during fermentation, possesses the ability to decontaminate aflatoxins B_1_ by converting it into aflatoxicol. In the same vein, *Rhizopus oligosporus*, when cultured with aflatoxin B_1_-synthesizing *A. flavus*, inhibited the production of the aflatoxin B_1_ and triggered the degradation^105^.

Fermentation was reported to reduce phytate contents in cereals, legumes, and tubers due to the activity of the endogenous phytase during the process. Fermentation of tempe with *Rhizopus oligosporus* decreased the phytate content to between 32.9–54.5% of the levels in control samples. As reported by Reddy and Pierson^48^ a decrease in phytate levels (48.5–96.3%) in fermented peanut press cake was obtained when fermented with either *Rhizopus oligosporus* or *Neurospora sp;* fermentation of cooked traditional beans with *Rhizopus oligosporus* led to a 47% decrease in trypsin inhibitory effects; cassava fermentation (48 h) resulted into reduced HCN amounts to <30% of the initial amounts.

### Lactic acid bacteria (LAB) fermentation

Lactic acid bacteria are the most promising bacteria used as fungal antagonists in food fermentation. Application of LAB in indigenous ffs have been in existence from ancient times, and they have gained the status of QPS (Qualified Presumption of Safety) and GRAS (Generally Regarded as Safe) by the European Food Safety Authority (EFSA) and the American Food and Drug Agency, respectively^106^. So far, many strains of LAB have been considered as “green preservatives” due to their ability to suppress fungal growth in food^107^. Various antifungal compounds including bacteriocins, organic acids, diacetyl, fatty acids, hydrogen peroxide, lactones, alcohols, bioactive antimycotic oxide, and reuterin have been found to be synthesized by different LABs^108^. The function of LAB strains is not restricted to fungal growth inhibition, and some are known to bind to fungal mycotoxins, leading to their inactivation.^109^

Inhibition of mycotoxins by LAB can take place through different mechanisms such as inhibition of productions of mycotoxins (by direct impact on fungal growth^110^, mycotoxin production inhibition via the modification of external environment^111,112^, and mycotoxin elimination through adsorption by LAB strains). Adsorption of mycotoxins through their cell wall components is the mechanism mostly common in the removal of mycotoxin by LAB from food matrices. Lactic acid bacteria have been proven to adsorb mycotoxins based upon the functional groups of their cell walls. For example, the cell wall of LAB as with other Gram-positive bacteria is made up of a thick complex peptidoglycan sacculus, enclosed by a cytoplasmic membrane, which is enriched with proteins, polysaccharides, lipoteichoic acids, and teichoic acids. Peptidoglycans and polysaccharides are known to be the major components of LAB for the removal of mycotoxins, and variations in the mycotoxin binding capacity of LAB species is due to the differences that exist in the peptidoglycan structure of their cell wall as well as the amount of available binding sites^113^. Mechanisms explaining the binding of LAB strains to different mycotoxins that are present in ffs are discussed in the subsequent subsections.

### Aflatoxins

**Fig. 5:**
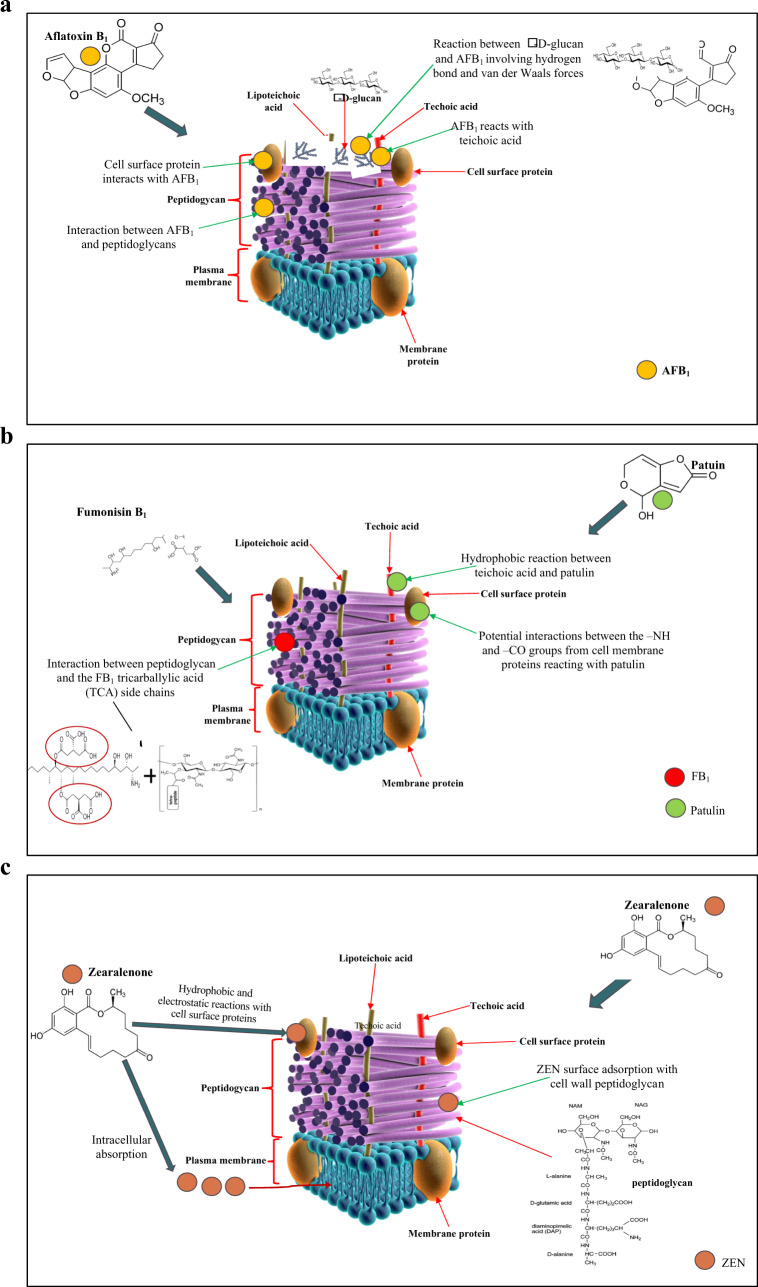
Diagrams illustrating possible interactions between some selected mycotoxins and bacteria cell wall components. **a** The Mechanisms of interactions between aflatoxin B1 with bacterial cell. Wall components such as peptidoglycan, cell wall teichoic acids, *β*-D-glucan, and cell surface proteins. Illustrations adapted from Adobe stock images, copyright Kateryna_Kon (http://stock.adobe.com/) under Standard Licensing. **b** The Mechanisms of interactions between fumonisin B1 and patulin with bacterial cell wall components. fumonisin B1 only reacts with cell wall peptidoglycan (interaction between tricarballyllic acid (TCA) of fumonisin B_1_ and peptidoglycans). The known possible mechanism for bacterial binding to patulin involves interaction with cell surface proteins (-NH or -CO groups). Illustrations adapted from Adobe stock images, copyright Kateryna_Kon (http://stock.adobe.com/) under Standard Licensing. **c** The Possible mechanisms of interactions between zearalenone and a LAB strain; interaction with intracellular proteins, reaction with cell wall peptidoglycans, or interaction with cell surface proteins (by hydrophobic or electrostatic reaction). Illustrations adapted from Adobe stock images, copyright Kateryna_Kon (http://stock.adobe.com/) under Standard Licensing. AFB Aflatoxin B1, ZEN Zearalenone, OTA Ochratoxin A, DON Deoxynivalenol, FB_1_ Fumonisin B_1_.

Figure 5a outlines all possible mechanisms for the binding of aflatoxin B_1_ to the cell wall components of LAB. The binding of *Lactobacillus rhamnosus* to aflatoxin B_1_ is associated primarily with cell wall peptidoglycans and without the role of cell wall proteins, exopolysaccharides, lipids, and minerals^114^. In contrast, teichoic acids besides peptidoglycans were also observed to be responsible for the binding of aflatoxin B_1_ by *Lactobacillus reuteri* NRRL14171 and *Lactobacillus casei* Shirota^115^. Furthermore, it has been studied that aflatoxin B_1_ can bind to cell *β*-d-glucans on walls of bacteria involving hydrogen bonds and van der Waals interactions^116^. Environmental conditions play a key role in the modulation of the number of binding sites available for mycotoxins, bacterial cell wall structure, and physiochemical characteristics^112^. Acid treatments were reported to increase the hydrophobic interactions because of the denaturing of the cell wall proteins, thereby increasing the number of binding sites to aflatoxin B_1_^117^. A better theoretical model was established by Bueno, et al.^118^ for aflatoxin B_1_ adsorption to LAB species. According to the authors, the amount of the mycotoxin adsorbed depends on the number of the available binding sites on the cell wall of a bacterium and the equilibrium constant^119^. However, this could be altered due to chemical, physical, or genetic changes.

### Ochratoxin A

Ochratoxin A has been found to bind to the cell wall components of *L. planetarium*, *Lactobacillus sanfranciscensis*, and *L. brevis* though the binding was shown to be altered not only by the hydrophobicity of the cell walls but also by the electro donor-acceptor and Lewis’s acid-base reaction^120^. In addition, the ability of LAB species to enhance mycotoxin binding was reported to be further improved the mutagenesis/genetic control or via the supplementation of some binding-promoting compounds in few cases.

### Fumonisins

Figure 5b shows the likely interactions between fumonisin B_1_ and bacterial cell wall components for mycotoxin removal or decontamination. Cell components and functional groups that are responsible for the interactivity and adsorption of fumonisin B_1_ and fumonisin B_2_ by *Lactobacillus paraplantarum* were studied by Niderkorn, et al.^121^ and observed that peptidoglycan or some compounds closely linked to it are possibly responsible for fumonisins binding, and without the role of the cell wall lipids, proteins and polysaccharides. Peptidoglycans were also reported to exhibit the maximum capacity of binding to fumonisins by *Lactobacillus* Pentosus X8 and *L. plantarum* B7^122^. The mechanism of the interaction between fumonisins and the cell wall peptidoglycan of LAB stains is yet to be fully understood. Nonetheless, Niderkorn, et al.^121^ in his study, proposed that tricarballylic acid chains of fumonisins react with peptidoglycans during the process of binding.

### Patulin

Zoghi, et al.^123^ indicated the function of cell surface adhesion proteins as the key structures for patulin binding. Various factors associated with the bio-adsorption of patulin by deactivated LAB species were investigated to further understand the mechanism in a recent study by Wang, et al.^124^. These authors reported that patulin binding by LAB strains significantly increased by esterification and NaOH pretreatments and reduced by pretreatments using trypsin, iodate, periodate, and lipase. Furthermore, it was revealed that the peptidoglycans of bacterial cell walls do not function in this absorption to any great degree. The authors indicated the possibility of thiol, esters, and alkaline amino acids as the likely compounds responsible for the absorption. Some more novel insights into the mechanisms of the paulin binding by strains of LAB were provided by Wang, et al.^125^ where they demonstrated the importance of the cell volume and area, and proposed that the higher the cell volume and area, the greater patulin absorption. In addition, the proteins on the cell surface including the carbohydrate components of LAB cells (C–O groups, OH, and NH) were revealed as the likely cell wall components involved in the patulin adsorption. The potential mechanism for patulin adsorption by bacteria strains can be found in Fig. 5b. However, the precise mechanism including the type of reaction between the bacterial cell wall components and patulin is still to be fully understood.

### Zearalenone, T-2 toxin, and trichothecene mycotoxins

Król, et al.^126^ evaluated the kinetics of zearalenone binding by *Lactococcus lactis* species as a function of time that gave a better understanding of the factors that regulate zearalenone binding. The rate of adsorption of zearalenone by *L. lactis* reduced from 5.49 μg/mL/min during the stage of absorption (720 min), where about 88% of zearalenone was adsorbed, and to 0.15 μg/mL/min at the second stage of adsorption where equilibrium was attained in the system. The mechanism proposed for the zearalenone removal by strains of LAB involved its interactivity with the cell wall proteins, peptidoglycans, or absorption into the bacterial cell succeeding interaction with the intracellular proteins (Fig. 5c).

Correspondingly, *L. plantarum* strain 102 was reported to bind T-2 toxin on cell wall components. The binding to cell walls is the only well-known mechanism responsible for trichothecenes removal by strains of LAB^127^. As studied by Zhou, et al.^128^, the cell wall of *L. lactis* CAMT22361 was involved in the removal of T-2 toxins. Non-protein elements of the cell-extracellular section played a major role in the toxin removal followed by the protein components of the extracellular section.

The application of LAB in eliminating mycotoxins in ffs is a general practice in several industries related to foods. *Lactobacillus* spp., *Bifidobacterium* spp., *Propionibacterium* spp., and *Streptococcus* spp, *Lactococcus* spp., *Leuconostoc* spp., *Pediococcus* spp. strains were reported to be often used to remove mycotoxins in ffs, as they are well known for their resistance and ability to bind to toxins^4^. *Lactobacillus fermentum* OYB, *L. fermentum* RS2, *L. plantarum* MW, *L. plantarum* YO, *L. brevis* WS3, and *Lactococcus* spp. RS3 has been reported for its anti-AF synthesizing ability when isolated from fermented gruels. Interestingly, *L*. *plantarum* YO suppressed the food contaminating aflatoxin B_1_ including the G-synthesizing *Aspergillus* spp. in *vitro* studies. This however suggests the antagonistic ability of the *Lactobacillus* strain in preventing or reducing mycotoxins in ffs^109^. In fermented maize meal, *Streptococcus lactis* and *Lactobacillus delbrueckii* reduced the amounts of increased zearalenone and fumonisin B_1_. This result reveals that LAB-mediated fermented maize meal eliminated or reduced the mycotoxins including aflatoxin B_1_ during the process^112^. The addition of *L. casei* during fermentation of kefir greatly about 88.17% of aflatoxin M_1_. The LAB strain works together with a kefir starter culture to decrease aflatoxin M_1_ in the food product^129^. *Aspergillus oryzae* MAO103, *A. oryzae* MAO104 isolated from *Meju* effectively degraded AFB_1_ to 90% within 2 weeks and further inhibited the growth of aflatoxin B_1_ produced from *Aspergillus flavus*^130^.

### Packaging techniques

Food packaging is developed to hold, preserve and protect foods from contamination (environmental and microbial) and other impacts such as shocks, odor, temperature, dust, light, physical damage, and humidity^131^. In the absence of adequate protection, food will become unappetizing, lose nutritional value, and become unsafe for consumption. For standard food packaging, the required protection is based on the fragility and stability of the food, the target shelf life, and the chain of distribution^132^. Specific packaging such as effective and smart packaging technologies are essential for fermented foods to preserve the product attributes including flavor, color, and texture, as their fermentation process continues though at a lesser rate^133^. Fermented foods have the potential of becoming contaminated with degraded products or components due to product-package interactivity. These chemical and physical changes during the package-product interactions may result in the migration of external microbial and chemical contaminants into the food, impacting the organoleptic properties and decreasing the food quality.

Active packaging is one of the innovative packaging used in fermented foods as it makes it convenient to see, feel, read or smell the food characteristics, while intelligent and smart packaging involves the use of sensors/indicators for providing relevant information on the food status or its surrounding system^134^.

Modified atmosphere packaging^135^ is a technique that can control gas composition in the environment surrounding the food within a gas-impermeable packaging. For instance, wheat and rye bread artificially inoculated with various fungi species were packaged with 0%, 50%, 75%, or 100% CO_2_; 1% or 0.03% O_2_; or with O_2_ absorber and balanced with N_2_^136^. The MAP was more potent against fungal growth on the rye bread, as lesser fungi developed with the increased CO_2_. But for *P. roqueforti*, this main contaminant of rye bread was inhibited only in the presence of an O_2_ absorber. Also, *Penicillium roqueforti* and *A. flavus* were unable to grow in both 40% and 60% CO_2_ environments balanced with N_2_ and <0.5% O_2_, but weakly grew (about 30 mm in 30-day incubation) in 20% CO_2_^137^.

Fermented products properties, however, are still inadequately understood and some of these products are still packaged using traditional methods. Therefore, there is a need for the development of suitable packaging technology before the commercialization of ffs for enhanced shelf life quality^138^

### Controlled processing conditions

The quality of ff products could be enhanced through the improvement of the process of fermentation and modernization of the automation in food industries. Indeed, several foods manufactures ensure Good Manufacturing Practices (GMP) and Hazard Analysis Critical Control points (HACCP) which greatly reduces potential risks to public health^132^. On the other hand, indigenous ffs are spontaneously produced at household levels or by small factories usually under hygienically insufficient and unmanaged conditions. Hence, the risks afterward are severe, requiring more rigid hygienic standards^139^. The process of fermentation itself, if appropriately done, can enhance food safety and public health. This is because fermentation-related microorganisms will generally compete with other microbes, causing competitive exclusion of pathogenic microbes that cause spoilage. For example, inhibitory amounts of ethanol are synthesized during alcohol fermentation; microbes may also produce some inhibitory substances such as bacteriocins, mycosin, and diacetyl, acetaldehydes that may suppress the growth of pathogenic microbes. Lastly, some food fermentation cand involve the addition of salt, hops, sulfites, nitrates, nitrites, and some antimicrobial materials that can suppress the survival or growth of spoilage fungi^132^.

Indigenous ffs are often consumed as routine diets by many people in SEA. However, pathogenic microbes-producing mycotoxins and plant toxins contribute to the major causes of health issues in humans. This paper has summarized the main mycotoxins and plant toxins present in ffs, and the strategies used for their prevention and/or decontamination. Most substrates used in the production of ffs are a potential source of toxic substances that are detrimental to human health. For instance, cereals and legumes (soybean) are commonly contaminated with mycotoxins (ochratoxin A, aflatoxins, zearalenone, deoxynivalenol and fumonisin), possess plant toxins (cyanogenic glycosides that transforms into HCN during the fermentation process, phytates, saponins, oxalates, and enzyme inhibitors) and accommodate fungal pathogens (*Penicillium*, *Fusarium* and *Aspergillus* spp.).

Studies have demonstrated that the use of substrates that are free from microbial contaminations, specific LAB strains, improved fermentation, and processing conditions (especially washing, prolonged soaking, decanting, and milling), and application of appropriate packaging techniques can effectively mitigate or counteract the toxic substances in ffs. However, it is important to note that, a single method has not been proven 100% effective for ensuring safe food. Hence, more studies into the application of combined management strategies through the integration of numerous potential control methods are required for the safe consumption of indigenous ffs and their sustainability in the global food industry.

## Data Availability

We declare that data sharing does not apply to our manuscript. This is a review article with no data analyzed during the study.
